# Aquaporin‐4 deficiency reduces TGF‐β1 in mouse midbrains and exacerbates pathology in experimental Parkinson's disease

**DOI:** 10.1111/jcmm.14147

**Published:** 2019-01-25

**Authors:** Xue Xue, Weiwei Zhang, Jifeng Zhu, Xiaojun Chen, Sha Zhou, Zhipeng Xu, Gang Hu, Chuan Su

**Affiliations:** ^1^ Jiangsu Key Laboratory of Pathogen Biology, Department of Pathogen Biology and Immunology Nanjing Medical University Nanjing Jiangsu China; ^2^ Department of Nuclear Medicine Nanjing First Hospital, Nanjing Medical University Nanjing Jiangsu China; ^3^ Department of Pathogen Biology and Immunology Nanjing University of traditional Chinese Medicine Nanjing Jiangsu China; ^4^ Jiangsu Key Laboratory of Neurodegeneration, Department of Pharmacology Nanjing Medical University Nanjing Jiangsu China; ^5^ Department of Pharmacology Nanjing University of Chinese Medicine Nanjing Jiangsu China

**Keywords:** aquaporin‐4 (AQP4), Parkinson’s disease (PD), transforming growth factor‐β1 (TGF‐β1), α‐synuclein (α‐syn)

## Abstract

Aquaporin‐4 (AQP4), the main water‐selective membrane transport protein in the brain, is localized to the astrocyte plasma membrane. Following the establishment of a 1‐methyl‐4‐phenyl‐1,2,3,6‐tetrahydropyridine (MPTP)‐induced Parkinson's disease (PD) model, AQP4‐deficient (AQP4^−/−^) mice displayed significantly stronger microglial inflammatory responses and remarkably greater losses of tyrosine hydroxylase (TH^+^)‐positive neurons than did wild‐type AQP4 (AQP4^+/+^) controls. Microglia are the most important immune cells that mediate immune inflammation in PD. However, recently, few studies have reported why AQP4 deficiency results in more severe hypermicrogliosis and neuronal damage after MPTP treatment. In this study, transforming growth factor‐β1 (TGF‐β1), a key suppressive cytokine in PD onset and development, failed to increase in the midbrain and peripheral blood of AQP4^−/−^ mice after MPTP treatment. Furthermore, the lower level of TGF‐β1 in AQP4^−/−^ mice partially resulted from impairment of its generation by astrocytes; reduced TGF‐β1 may partially contribute to the uncontrolled microglial inflammatory responses and subsequent severe loss of TH^+^ neurons in AQP4^−/−^ mice after MPTP treatment. Our study provides not only a better understanding of both aetiological and pathogenical factors implicated in the neurodegenerative mechanism of PD but also a possible approach to developing new treatments for PD via intervention in AQP4‐mediated immune regulation.

## 1 INTRODUCTION

Parkinson's disease (PD) is a common neurodegenerative disorder and the most common movement disorder. To date, approximately 2% of the population over the age of 65 is affected worldwide.^1^, ^2^, ^3^ Prominent clinical features of PD are motor symptoms (bradykinesia, tremor, rigidity and postural instability) and non‐motor‐related symptoms (olfactory deficits, autonomic dysfunction, depression, cognitive deficits and sleep disorders).^4^ Neuropathological hallmarks of PD are the loss of dopaminergic neurons (DNs) in the substantia nigra of the midbrain and intracellular inclusions containing α‐synuclein (α‐syn) called Lewy bodies.^5^

The aetiologies of PD remain poorly understood. Although host genetics and environmental factors affect the onset and progression of PD,^6^, ^7^ epidemiologic, animal, human and therapeutic studies all support a critical role for a neuroinflammatory cascade in the disease (eg microglial activation and an increase in astroglia).^8^, ^9^, ^10^ and suggest that activated microglia are the primary mediators in neuroinflammatory responses.^11^, ^12^ Activated microglia are commonly seen within the substantia nigra pars compacta (SNpc) of PD brains investigated at autopsy; these cells directly induce significant, highly detrimental neurotoxic effects by excessive production of a large array of cytotoxic factors such as interleukin‐1β (IL‐1β), tumour necrosis factor‐α (TNF‐α), interleukin‐6 (IL‐6) and nitric oxide (NO).^13^, ^14^, ^15^ Moreover, as an antigen‐presenting cell (APC), activated microglia express costimulatory molecules, such as cluster of differentiation 40 (CD40), cluster of differentiation 80 (CD80; B7‐1) and cluster of differentiation 86 (CD86; B7‐2), that promote APC‐dependent T‐cell activation.^16^, ^17^, ^18^ Subsequently, activated T cells injure neurons by cell contact‐dependent mechanisms that involve Fas ligand (FasL) and/or release of cytotoxic factors.^19^ Attenuation of microglial activation can protect up to 90% of DNs in PD animal models.^20^, ^21^, ^22^

Aquaporin‐4 (AQP4), originally known as a mercurial‐insensitive water channel, is most strongly expressed in astrocytes throughout the brain and spinal cord, as well as in ependymal cells lining the brain ventricles; it is involved in the regulation of water homeostasis in the brain.^23^, ^24^, ^25^ Recently, AQP4 expression has been reported to be involved in the pathology of the development of PD in a 1‐methyl‐4‐phenyl‐1,2,3,6‐tetrahydropyridine (MPTP)‐induced mouse model. In our previous studies, compared with AQP4^+/+^ mice, AQP4^−/−^ mice were significantly more prone to MPTP‐induced neurotoxicity and subsequently exhibited significantly stronger microglial responses in the midbrain and more severe PD symptoms.^26^, ^27^, ^28^ However, the mechanisms underlying hyperactive microglial responses and more severe clinical symptoms in PD after administration of MPTP in AQP4‐deficient mice remain unclear.

In this study, significantly decreased transforming growth factor‐β1 (TGF‐β1) led to increased neuroinflammatory responses in the midbrain and more severe PD pathology in AQP4‐deficient mice after MPTP intoxication. Our findings suggest a novel role for AQP4 in brain neurodegeneration and an opportunity for the development of new therapeutic approaches to treat neurodegenerative diseases.

## 2 MATERIAL AND METHODS

### 2.1 Transgenic mice

AQP4‐deficient mice were generated as previously described.^27^ AQP4^−/−^ mice were maintained on the CD1 background. AQP4^+/+^ CD1 mice were used as wild‐type (WT) control animals. Mice were identified by polymerase chain reaction (PCR) analysis of tail samples at post‐natal day 5 and by Western blot analysis of the cerebral cortex. Mice were bred and maintained under environmentally controlled conditions (ambient temperature, 22°C; humidity, 40%) on a 12‐hour light/dark cycle with access to food and water. All experiments were performed on age‐ and weight‐matched littermates (20‐28 g). Mouse breeding was performed to achieve timed pregnancy with an accuracy of ±0.5 days.

All experiments were approved by IACUC (Institutional Animal Care and Use Committee of Nanjing Medical University). All efforts were made to minimize animal suffering and to reduce the number of animals used for the experiments.

### 2.2 Acute MPTP treatment

Sixteen‐week‐old male AQP4^+/+^ and AQP4^−/−^ mice were injected intraperitoneally (i.p.) four times with MPTP‐HCl (20 mg/kg of free base; Sigma Chemicals, St. Louis, MO) in saline or saline alone for controls at 2‐hour intervals. The total dose per mouse was 80 mg/kg, and the mice were killed 7 days after the last injection. Mortality rates in acute MPTP‐treated AQP4^−/−^ mice (41%, 35/85) were twofold higher than in AQP4^+/+^ mice (21%, 17/80, *P* < 0.05). As the mice of MPTP group underwent an obvious mortality after MPTP treatment, only mice that went through all 7 days procedure were included in the following statistical analyses. MPTP handling and safety measures were in accordance with published guidelines.^29^

### 2.3 Chronic MPTP/probenecid treatment

Sixteen‐week‐old male AQP4^+/+ ^and AQP4^−/− ^mice received a total of 10 doses of MPTP‐HCl (20 mg/kg in saline, subcutaneously [sc]) in combination with an adjuvant, probenecid (250 mg/kg in dimethyl sulfoxide [DMSO], ip). Mice were treated in a similar manner with probenecid and saline as controls. The 10 doses were administered on a 5‐week schedule, such that injections were given with an interval of 3.5 days between consecutive doses. Animals were killed 7 days after the last injection. AQP4^−/−^ and AQP4^+/+^mice after chronic MPTP treatment showed no mortality. Probenecid was purchased from Sigma Chemical Co. Probenecid was used to inhibit the rapid clearance and excretion of MPTP from the brain and kidney following each injection. Neither probenecid nor DMSO at the concentrations used in this study produced any significant effect on striatal dopamine (DA) contents.^30^

### 2.4 Mesencephalic primary neuron culture and treatment

Primary mesencephalic neuronal cultures were prepared from the ventral mesencephalon of gestational 16‐ to 18‐day‐old AQP4^+/+^ and AQP4^−/−^ mouse embryos. Mesencephalic tissues were dissected and maintained in ice‐cold Ca2^+^‐free Hanks’ Balanced Salt Solution (HBSS) (Gibco, Grand Island, NY) and then dissociated in HBSS containing trypsin‐0.25% ethylenediaminetetraacetic acid (EDTA) for 20 minutes at 37°C. Dissociated cells were then plated at equal density (2 × 10^6 ^cells) in a 25‐cm^2^ flask precoated with 1 mg/mL poly‐d‐lysine (Sigma). Cultures were maintained in a chemically defined medium consisting of neurobasal medium fortified with B‐27 supplements, 500 μg/mL of glutamine, 100 IU/mL penicillin and 100 μg/mL streptomycin (Invitrogen, Carlsbad, CA). The cells were maintained in a humidified CO_2_ incubator (5% CO_2_ and 37°C) for 24 hours. Half of the culture medium was replaced every 2 days. Seven‐day‐old cultures were used for the experiments. Primary mesencephalic DNs were exposed to 10 μmol/L MPP^+^ (Sigma) for 24 hours.

Primary cultures of mixed glia from day 1‐2 newborn mice were prepared. Briefly, following the removal of meninges, brain tissues were minced and incubated in a rocking water bath at 37°C for 30 minutes in HBSS (Gibco) in the presence of 0.25% trypsin (Sigma). Enzyme‐digested dissociated cells were triturated with 0.25% of foetal bovine serum (FBS, Gibco), followed by a wash and centrifugation (300× *g* for 10 minutes). The pellet was resuspended in HBBS and passed through 100‐μm nylon mesh, followed by a second wash and centrifugation (300× *g* for 10 minutes). Following dilutions with astrocyte‐specific medium (Dulbecco's Essential Medium containing 1% penicillin‐streptomycin, 10% FBS), the cells were plated and allowed to adhere for 1 day in a humidified CO_2_ incubator at 37°C. After 24 hour, any non‐adherent cells were removed, and fresh astrocyte‐specific medium was added. Adherent cells were maintained in astrocyte‐specific medium for 10 days with a medium change every 3‐4 days. The microglia population peaked at 12‐14 days in these cultures. Microglia‐enriched cultures were thoroughly agitated in an orbital incubator shaker (250 rpm for 2 hours at 37°C) to remove any cells adherent to the astrocyte monolayer. Immediately following agitation, all cells suspended in the culture medium were collected and centrifuged at 300× *g* for 5 minutes at 4°C. The cell pellet contained microglia that were resuspended and diluted with fresh astrocyte‐specific medium, bringing the cells to a final concentration of 8 × 10^5^ cells/mL until assayed. The original flasks in which the microglia had been shaken were maintained with astrocyte‐specific medium for subsequent experiments. Primary astrocytes were seeded at 1 × 10^6 ^cells per well in 6‐well plates and incubated with phosphate buffered saline (PBS) or MPP^+^ (50 μmol/L) for 48 hours in 0.1% serum‐supplemented medium. The culture medium was collected and centrifuged at 300 *g* for 5 minutes, then the volume of each supernatant was adjusted to the same volume (to standardized preparations) and immediately stored at −80°C until used for TGF‐β1 assay by ELISA using commercial kits.

### 2.5 BV‐2 cell culture

The immortalized microglial cell line BV‐2, derived from raf/myc‐immortalized murine neonatal microglia, was kindly provided by Prof. Gang Hu. BV‐2 cells were incubated under humidified 5% CO_2_ and 95% O_2_ at 37°C in Dulbecco's Modified Eagle's Medium (DMEM, Gibco) medium containing 10% FBS and 1% streptomycin and penicillin (Gibco).

### 2.6 Brain homogenate preparation

Mice were sacrificed 7 days after either MPTP injection or TGF‐β1 injection under deep anaesthesia with chloral hydrate. The midbrain was immediately removed from the brain and homogenized in iced PBS (ratio: midbrain tissues from five mice: 200 μL PBS). Protein concentrations were determined by the Bradford method. The supernatant of the tissue homogenate was collected, subpackaged and stored (at −80°C) for the following incubation with BV‐2 cells. The incubation concentration was 50 μg/mL.

### 2.7 TGF‐β1 and anti‐TGF‐β1 treatment in vitro

AQP4^+/+^ or AQP4^−/−^ mouse brain homogenate was used to activate BV‐2 cells in vitro. Before in vitro activation, BV‐2 cells in the AQP4^−/−^ group were pre‐treated with purified recombinant human TGF‐β1 (rhTGF‐β1, 240B, R&D, and UK) for 1 hour, while BV‐2 cells in the AQP4^+/+ ^group received anti‐TGF‐β1 (1 μg/mL, T8250‐16A, USBiological, Salem, MA) pre‐treatment for 1 hour. BV‐2 cells in medium without TGF‐β1/anti‐TGF‐β1 served as controls.

### 2.8 TGF‐β1 administration in vivo

AQP4^+/+^ and AQP4^−/−^ mice were injected i.p. four times with MPTP‐HCl in saline at 2‐hour intervals, and the total dose per mouse was 80 mg/kg. After 24 hours, the mice were anaesthetized with 3% chloral hydrate (Sigma). After anaesthesia, the animals were placed in a stereotaxic apparatus (Stoelting Instruments, Wood Dale, IL). Unilateral injection of rhTGF‐β1^31^ (2 μg rhTGF‐β1 in 100 μL sterile vehicle (saline containing 0.1% bovine serum albumin and 4 mmol/L HCl) was performed in the left striatum (coordinates from the bregma: AP, +0.5 mm; ML, +2.0 mm; DV1, 3.6 mm, DV2, 3 mm) with a Hamilton syringe (0.46 mm in diameter) at a rate of 0.25 μL/min. The needle was left in place for 3 minutes after the injection. Then, the needle was slowly moved 0.6 mm to the second injection position (DV2, 3 mm). The total injection volume was 2.5 μL, and the needle was left in place for 3 minutes after injection. Then, the needle was slowly removed to prevent reflux. Saline‐lesioned mice were injected with 2.5 μL of sterile vehicle (saline containing 0.1% bovine serum albumin and 4 mmol/L HCl) into the left striatum and served as controls. After injection, the mice were kept in cages with a constant temperature (25°C) and humidity. They were exposed to a 12:12‐hour light‐dark cycle and had unrestricted access to tap water and food. Mice were killed for further study at 6 days after the MPTP injection.

### 2.9 Flow cytometry

After treatment, BV‐2 cells were incubated with 5 mmol/L EDTA/PBS for 10 minutes at 37°C, and then detached by gentle pipetting to prepare single cell suspension. Cells were incubated with Fc Block (anti‐mouse CD16/CD32 antibody, BD Pharmingen) at 0.125 μg/10^6^ cells in 1× phosphate‐buffered saline containing 1% FBS and then maintained on ice for 30 minutes. The following PE‐conjugated mAbs (all purchased from eBioscience, CA) were used at 1:100 dilution: PE‐conjugated rat anti‐mouse MHCII (IgG2b), PE‐conjugated rat anti‐mouse CD80 (IgG2b), PE‐conjugated rat anti‐mouse CD40 (IgG2b). The background fluorescence was evaluated by staining the cells with isotype matched PE‐conjugated rat IgG2b. At the end of incubation, cells were washed three times with PBS. Cells were centrifuged and resuspended in 0.3 mL PBS and analysed on FACSCalibur flow cytometer (BD Biosciences, San Diego, CA) and CellQuest software (BD Biosciences); data were collected on 10 000 cells per condition.

### 2.10 Immunohistochemistry staining

At the end of the experiments, the mice were perfused with 4% paraformaldehyde (PFA, Sigma). Brain samples were collected and post‐fixed in 4% PFA at 4°C overnight. They were transferred to 15% sucrose in PBS overnight and then to 30% sucrose overnight until the brain sunk to the bottom of the tube. Forty‐micrometre sections were incubated overnight and mounted on the poly‐L‐lysine coated slides. Sections were incubated with rabbit anti‐TH antibodies (1:1000; T8700; Sigma), rabbit anti‐glial fibrillary acidic protein (GFAP) antibodies (1:1000; AB5804; Millipore), or rat anti‐mouse Mac‐1 polyclonal antibodies (1:50 dilution, CD11b; AbD Serotec, Oxford, UK) for the detection of TH, GFAP and microglia. Next, the sections were incubated for 1 hour with secondary antibodies. Immunostaining was visualized by incubation with a 3,3′‐diaminobenzidine (DAB) kit [EnVision+ Dual Link System‐horseradish peroxidase (HRP) (DAB+); Dako, Carpinteria, CA]. Sections were then counterstained with thionin. Control staining was performed without the primary antibodies. The total numbers of tyrosine hydroxylase‐positive cells (TH^+^) neurons, GFAP^+^ and Mac^+^ cells in the SNpc were obtained stereologically using the optical fractionator method.^32^ For cell quantification in in vivo studies, the number of TH^+^ in the SNc of the midbrain was assessed using the optical fractionator (Stereo Investigator 7, MBF bioscience, Williston, VT). Briefly, the regions of SNc in the midbrain sections were outlined at low magnification (40×).For TH^+^ cells, the counting frame size was 50 × 50 μm and the sampling grid size was 100 × 100 μm. All stereological analyses were performed under the 200× magnification of an Olympus BX52 microscope (Olympus America Inc, Melville, NY). Within one counting frame, positive cells counted must show both TH staining in the cell body and blue staining in the nuclei, and the nuclei does not touch or cross the red avoidance lines of the counting frame. The sampling scheme was designed to have coefficient of error (CE) less than 10% in order to get reliable results. The total number of TH^+^ neurons in entire extent of SNc was counted from five mouse brains per group. Each brain contains 12 serial sections at a three intervals. The stereologer was blinded to the person analysing the histology and treatment groups for each experiment.

For colocalization analysis, sections were immunolabelled overnight with mouse monoclonal anti‐α‐syn (1:500; BD, Biosciences, San Diego, CA) and rabbit anti‐TH antibodies (1:1000; T8700; Sigma) or rabbit anti‐GFAP antibodies (1:1000; AB5804; Millipore), followed by incubation with fluorescein isothiocyanate‐conjugated goat anti‐mouse secondary antibodies (1:500) and PE‐Cy5 red‐conjugated goat anti‐rabbit antibodies (1:500) for 1 hour. Immunostaining was visualized with fluorescence microscopy (LSM5 PASCAL, Carl Zeiss, Oberkochen, Germany). Quantification of GFAP and Iba1 staining was performed by obtaining optical density measurements using the Image Quant 1.43 software (NIH) and corrected against background signal levels.

### 2.11 Quantitative real‐time PCR

Under deep anaesthesia, saline or MPTP‐injected AQP4^+/+^ or AQP4^−/− ^mice were killed by decapitation. The midbrain was removed for RNA isolation. Primary cells (neurons, astrocytes and microglia) and a cell line (BV‐2) were collected by centrifugation, and total RNA was extracted using a RNeasy mini kit (Qiagen, Valencia, CA) according to the manufacturer's protocol. First‐strand cDNA was synthesized from 1 mg of total RNA by SuperScript II reverse transcriptase (Invitrogen). The primer sequences were 5′‐GTGGTTCATGGAGTGACAAC‐3′ (forward) and 5′‐AGGCTTCAGGCTCATAGTCT‐3′ (reverse) for α‐syn, 5′‐CAACCAACAAGTGATATTCTCCATG‐3′ (forward) and 5′‐GATCCACACTCTCCAGCTGCA‐3′ (reverse) for IL‐1β, 5′‐CATCTTCTCAAAATTCGAGTGACAA‐3′ (forward) and 5′‐CATCTTCTCAAAATTCGAGTGACAA‐3′ (reverse) for TNF‐a, 5′‐GAGGATACCACTCCCAACAGACC‐3′ (forward) and 5′‐AAGTGCATCATCGTTGTTCATACA‐3′ (reverse) for IL‐6. 5′‐TATGCTAAAGAGGTCACCCGC‐3′ (forward) and 5′‐ACCAAGGTAACGCCAGGAATT‐3′ (reverse) for TGF‐β1. 5′‐GTTTCTTACTCCTTGGAGGCCAT‐3′ (forward) and 5′‐TGATGACATCAAGAAGTGGTGAA‐3′ (reverse) for glyceraldehyde 3‐phosphate dehydrogenase (GAPDH).

Relative quantification of gene expression was performed with an Applied Biosystems 7300 Real‐Time PCR system (Warrington, UK). The reaction mixture (20 μL) for PCR consisted of 1 mL cDNA template and 9 μL water, 8 μL FastStart Universal SYBR Green Master (ROX, Roche, Mannheim, Germany) and 2 μL of primer. Each PCR cycle consisted of denaturation at 95°C for 5 minutes, 50 cycles of 20 seconds at 95°C and 45 seconds at 60°C. Polymerase chain reaction amplification was carried out for 40 cycles. The mRNA expression was quantified by the 2^−ΔΔCt^ method.

### 2.12 Enzyme‐linked immunosorbent assay for IL‐1β, TNF‐A, IL‐6 and TGF‐β1 in serum and cell culture supernatant

Serum samples were isolated from saline or MPTP‐injected AQP4^+/+^ or AQP4^−/−^ mouse peripheral blood. TGF‐β1 enzyme‐linked immunosorbent assay (ELISA) kits (R&D Systems, Minneapolis, MN) were used to analyse serum samples according to the manufacturer's instructions. The levels of IL‐1β, TNF‐a and IL‐6 in BV‐2 cell culture supernatants were determined using specific ELISA kits (BioLegend, San Diego, CA) according to the manufacturer's instructions.

### 2.13 Western blot assays

Cells were washed twice with PBS after treatment and solubilised in radioimmunoprecipitation assay (RIPA) lysis buffer (Cell Signaling, Danvers, MA). Tissue samples were homogenized in RIPA lysis buffer. Protein concentrations were determined by the Bradford method. Protein samples (30 μg) were separated by 10% SDS‐PAGE and transferred onto a nitrocellulose membrane (Amersham Biosciences, Piscataway, NJ). After blocking in 10% milk with Tris‐buffered saline‐Tween‐20 (TBS‐T) buffer (10 mmol/L of Tris‐HCl, 120 mmol/L of NaCl, 0.1% Tween‐20, pH 7.4) for 1 hour at room temperature, the membrane was incubated with mouse anti‐rat α‐syn (1:500; BD), mouse monoclonal β‐actin (1:10000; Sigma), or rabbit anti‐TGF‐β1 antibodies (1:1000, T8250‐16A; USBiological). Membranes were then washed three times in TBS‐T buffer, followed by incubation with HRP‐conjugated anti‐rabbit/mouse IgG (1:10000) at room temperature for 1 hour and washed three times in TBS‐T. Visualization was carried out using an enhanced chemiluminescence (ECL) kit (GE Healthcare, Bucks, UK). The density of the bands on Western blots was quantified by densitometric analysis of the scanned blots using ImageQuant software (Bio‐Rad, Hercules, CA, USA). The relative phosphorylation was normalized to total protein.

### 2.14 Statistical analysis

Data analyses and graphs were performed using spss software for Windows version 16.0 (SPSS Inc, Carson City, NV). For mRNA expression, ELISA and immunohistochemistry analysis, comparisons were performed by two‐way ANOVA followed by a Newman‐Keuls post‐hoc multiple comparison test. The results were expressed in the text as the mean ± SEM, and statistical significance was established with a *P* value ≤0.05.

## 3 RESULTS

### 3.1 Brain homogenates from MPTP‐treated AQP4^−/−^ but not AQP4^+/+^ mice induced stronger activation in the microglia cell line BV‐2

**Figure 1:**
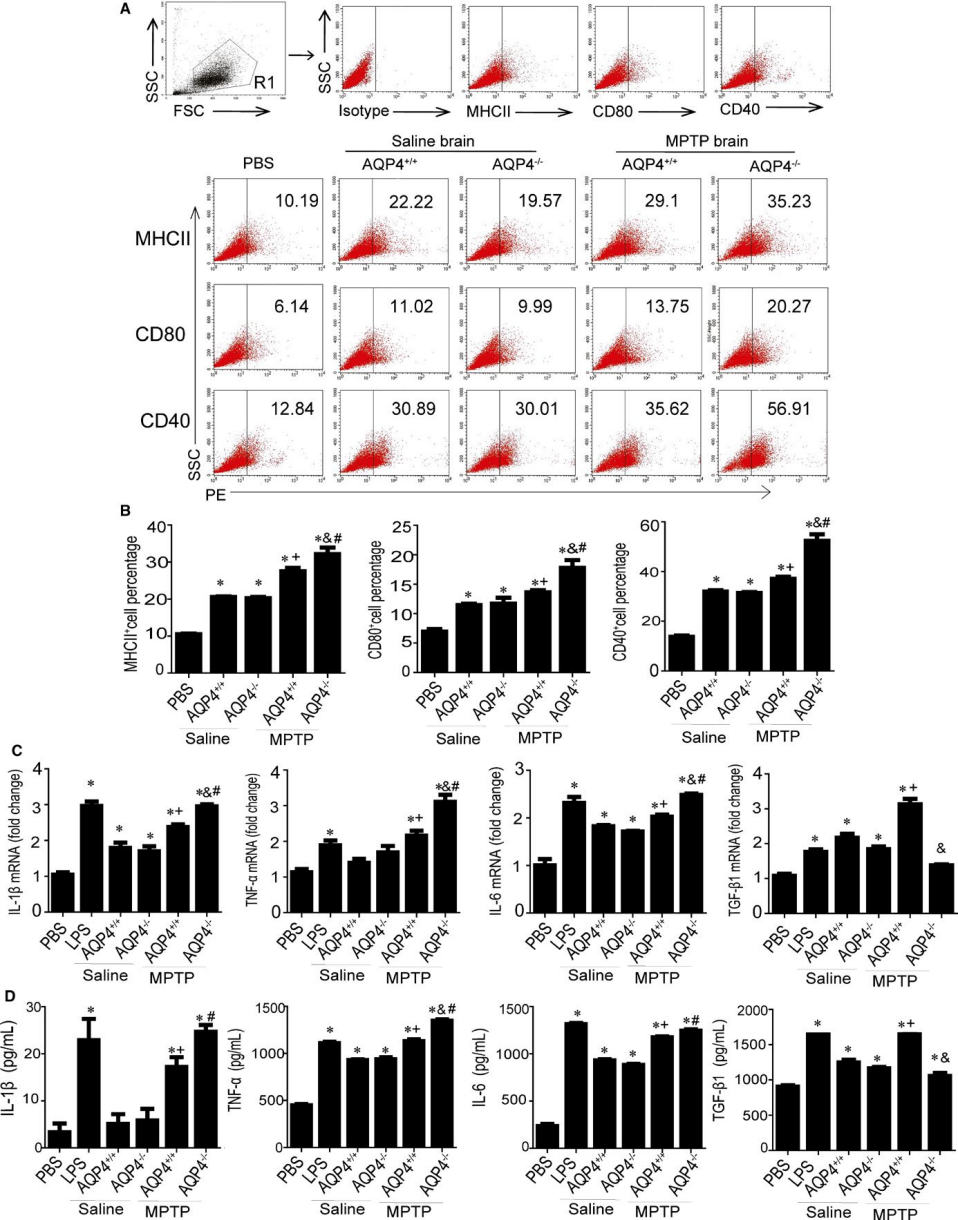
Brain homogenate‐induced expression of costimulatory molecules and inflammatory factors in BV‐2 cells. BV‐2 cells were maintained in control medium, lipopolysaccharide (LPS) 500 ng/mL, or brain homogenates (50 μg/mL) from saline‐ or 1‐methyl‐4‐phenyl‐1,2,3,6‐tetrahydropyridine (MPTP)‐injected AQP4^+/+^ or AQP4^−/−^ mice for 24 h. A, Cells were separately stained with anti‐MHCII‐PE, anti‐CD80‐PE, and anti‐CD40‐PE. FACScan profiles from a representative experiment are shown. B, Histograms are expressed as the percentage of MHCII (CD80 or CD40) positive cells in the total cell population. Representative histograms were obtained by flow cytometry analysis. C, interleukin‐1β (IL‐1β), tumour necrosis factor‐α (TNF‐α), interleukin‐6 (IL‐6) and transforming growth factor‐β1 (TGF‐β1) mRNA levels were determined with Quantitative real‐time PCR (Qrt‐PCR).The transcript levels for each cytokine in BV‐2 cells were normalized to the level in BV‐2 treated with phosphate buffered saline (PBS). D, Protein levels were determined by ELISA. Data were from five mice per group and are representative of three independent experiments. *P < 0.05 compared with PBS; +*P* < 0.05 compared with saline‐injected AQP4^+/+^ mice; #*P* < 0.05 compared with saline‐injected AQP4^−/−^ mice; &*P* < 0.05 compared with MPTP‐injected AQP4^+/+^ mice

In our previous study, administration of the neurotoxin MPTP resulted in more severe microgliosis and remarkably greater losses of TH^+^ neurons in the SNpc in AQP4^−/−^ mice.^26^, ^28^, ^33^, ^34^ However, AQP4 was not expressed on macrophage receptor 1 (Mac‐1^+^) microglia,^28^, ^35^, ^36^, ^37^ potentially excluding the possibility of AQP4 deficiency directly influencing microglial responses. Thus, to investigate whether any possible factor(s) in the brain might be responsible for differences in microgliosis between AQP4^+/+^ and AQP4^−/−^ mice after MPTP treatment, we stimulated the microglia cell line BV‐2 for 24 hours with midbrain homogenates from MPTP‐treated AQP4^+/+ ^and AQP4^−/−^ mice. As shown in Figure 1A and B, compared with incubation with PBS treatment controls, incubation with brain homogenates from either saline‐ or MPTP‐injected AQP4^+/+^ or AQP4^−/−^ mice significantly induced increases in the expression of costimulatory molecules major histocompatibility complex II (MHCII), CD80, and CD40 in BV‐2. Compared with brain homogenates from saline‐treated AQP4^+/+^mice (or AQP4^−/−^ mice), brain homogenates from MPTP‐treated AQP4^+/+ ^mice (or AQP4^−/−^ mice) induced a much higher increase in the costimulatory molecules MHCII, CD80, and CD40. Similarly, compared with PBS treatment controls, treatment with brain homogenates from either saline‐ or MPTP‐injected AQP4^+/+^ or AQP4^−/−^ mice increased both mRNA (Figure 1C) and protein (Figure 1D) expression levels of pro‐inflammatory TNF‐α, IL‐1β, and IL‐6 cytokines in BV‐2. In addition, compared with brain homogenates from MPTP‐treated AQP4^+/+^ mice, brain homogenates from MPTP‐treated AQP4^−/−^ mice induced higher increases in TNF‐α but not in TGF‐β1 cytokines in BV‐2 cells. These results suggest that some factors in AQP4^−/−^ mouse brains facilitate the higher expression of pro‐inflammatory cytokines but lower levels of TGF‐β1 after MPTP treatment.

### 3.2 Neither AQP4^+/+^ nor AQP4^−/−^ astrocytes showed high α‐syn protein expression

**Figure 2:**
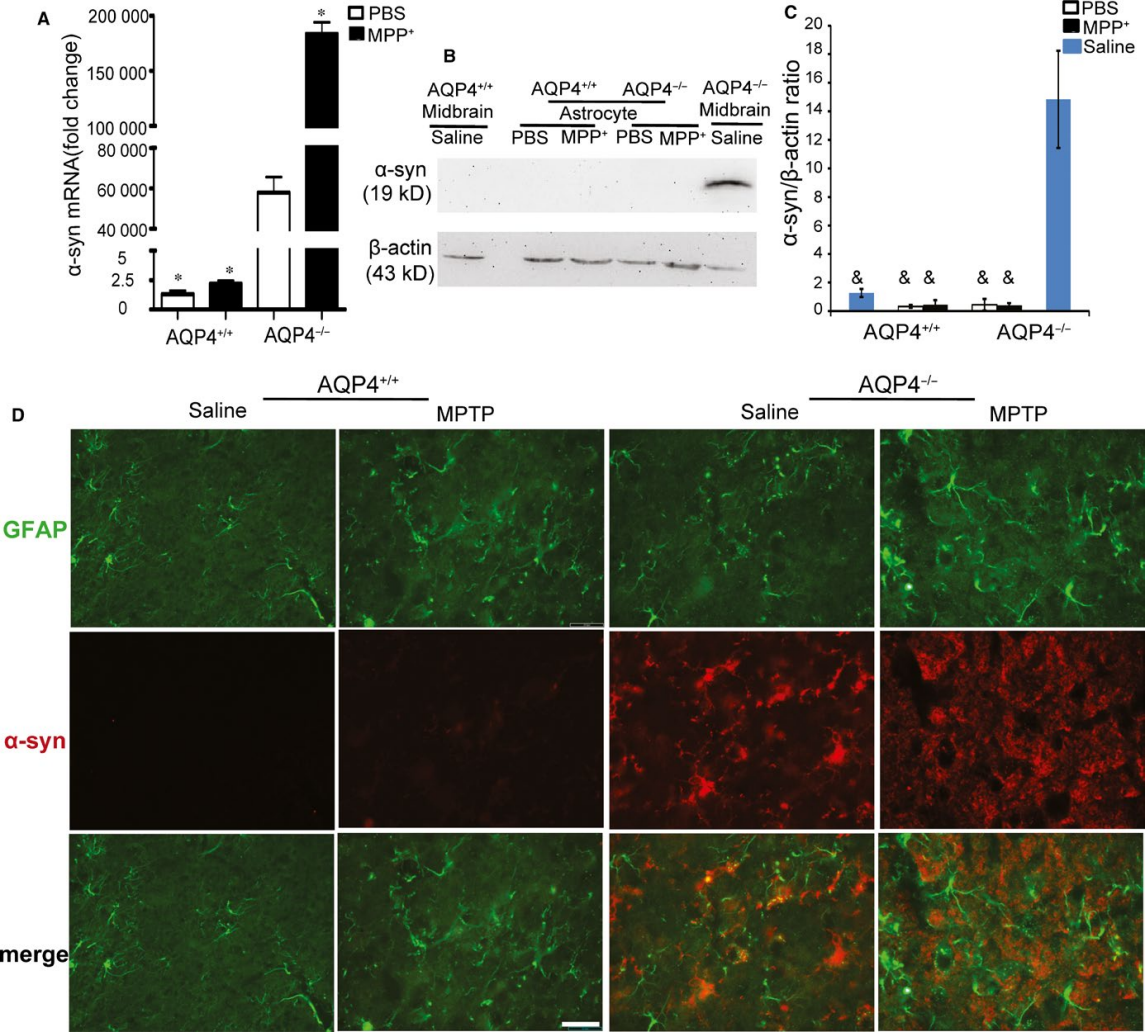
Differential expression of α‐synuclein (α‐syn) in AQP4^+/+^ or AQP4^−/−^ astrocytes. Analysis of α‐syn mRNA and protein expression in primary cultured midbrain astrocytes of AQP4^+/+^ or AQP4^−/−^ mice treated with phosphate buffered saline (PBS) or MPP^+^. A, Quantitative real‐time PCR (Qrt‐PCR) analysis of α‐syn mRNA expression 48 h after PBS or MPP^+^ (50 μmol/L) administration in astrocytes. B, Western blot analysis of α‐syn expression in PBS‐ or MPP^+^‐treated primary astrocytes. Saline‐treated AQP4^−/−^ mouse midbrains served as a positive control. Quantitative results (C) were obtained by measurement of the optical density of each band using a computerized image analysis system as described in Section 2. D, Representative photomicrographs illustrating double‐immunofluorescence staining visualized with confocal microscopy of α‐synuclein‐1 (red) and glial fibrillary acidic protein (GFAP) (green) in the substantia nigra pars compacta (SNpc) of saline or 1‐methyl‐4‐phenyl‐1,2,3,6‐tetrahydropyridine (MPTP)‐injected AQP4^+/+^ or AQP4^−/−^ mice. Scale bar = 50 μm. Data were from five mice per group and are representative of three independent experiments. *P < 0.05 compared with PBS‐treated AQP4^−/−^ astrocytes; &*P* < 0.001 compared with saline‐treated AQP4^−/−^ mouse midbrain

Previous studies suggested that α‐syn plays an important role in the initiation and maintenance of inflammation in PD patients and a MPTP‐PD animal model by activating microglial cells to produce TNF‐a and IL‐1β.^38^, ^39^, ^40^ Since astrocytes highly express AQP4,^41^ we next determined whether there were any differences in the expression of α‐syn between AQP4**^+/+^** and AQP4**^−/−^** mouse astrocytes after 1‐methyl‐4‐phenylpyridinium (MPP^+^, the active metabolite of MPTP) treatment. Quantitative reverse transcription‐polymerase chain reaction (qRT‐PCR) showed that AQP4^−/−^ astrocytes expressed much higher levels of α‐syn mRNA than did AQP4^+/+ ^astrocytes even under the control condition without MPP^+^ treatment. MPP^+^ treatment resulted in a further increase in α‐syn mRNA expression in AQP4^−/−^ astrocytes (Figure 2A). However, Western blotting showed no detectable levels of α‐syn protein in either AQP4^+/+^ or AQP4^−/−^ astrocytes regardless of treatment with or without MPP^+^ (Figure 2B,C).

Furthermore, midbrain sections of both AQP4^+/+^ and AQP4^−/−^mice were examined by GFAP (activated astrocytes) and α‐syn staining. AQP4^−/−^ and AQP4^+/+^ mouse midbrains exhibited similar GFAP staining without MPTP treatment. However, much stronger α‐syn staining was shown only in AQP4^−/−^ mouse midbrains with or without MPTP treatment. 1‐methyl‐4‐phenyl‐1,2,3,6‐tetrahydropyridine treatment resulted in significantly enhanced GFAP staining with a slight increase in α‐syn in the astrocytes of AQP4^−/−^ mouse midbrains. However, no co‐staining of GFAP and α‐syn was observed in the midbrains of both AQP4^+/+^ and AQP4^−/−^ mice with or without MPTP treatment (Figure 2D).

These results suggested that after MPTP treatment, AQP4 deficiency results in enhanced astrocyte activation but no detectable difference in α‐syn protein levels in astrocytes between AQP4^+/+^ and AQP4^−/−^ mice.

### 3.3 AQP4^−/−^ neurons expressed higher levels of α‐syn with or without MPP^+^ treatment

**Figure 3:**
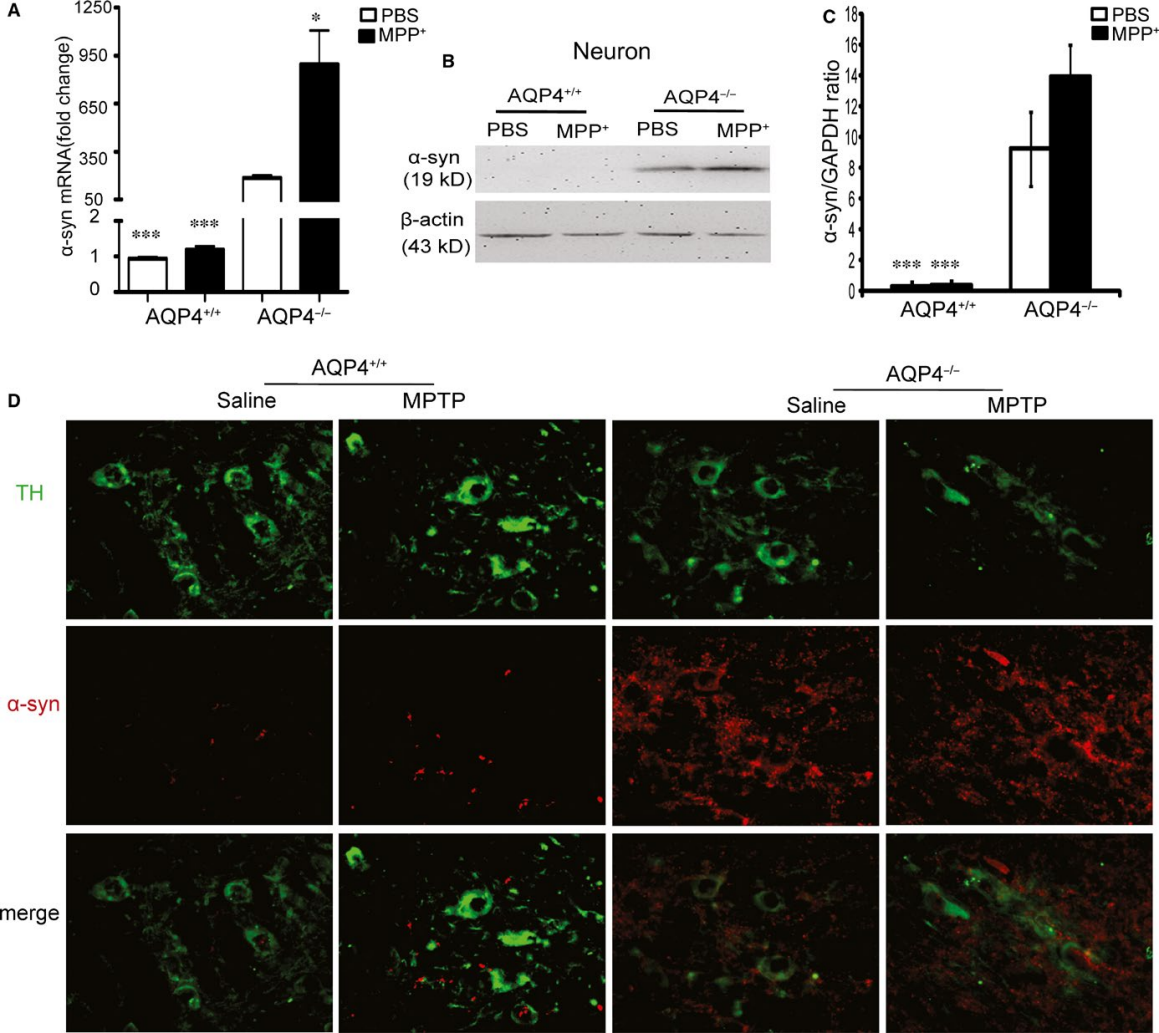
Differential expression of α‐synuclein (α‐syn) in AQP4^+/+^ or AQP4^−/−^ neurons. Analysis of α‐syn mRNA and protein expression in primary cultured midbrain neurons of AQP4^+/+^ or AQP4^−/−^ mice treated with PBS or MPP^+^. A, Quantitative real‐time PCR (Qrt‐PCR) analysis of α‐syn mRNA expression 24 h after PBS or MPP^+^ (10 μmol/L) administration in neurons. B, Western blot analysis of α‐syn expression in phosphate buffered saline (PBS)‐ or MPP^+^‐treated primary neurons. Quantitative results (C) were obtained by measurement of the optical density of each band using a computerized image analysis system as described in Section 2. D, Representative photomicrographs illustrating double‐immunofluorescence staining visualized with confocal microscopy of α‐synuclein‐1 (red) and tyrosine hydroxylase (TH) (green) in the substantia nigra pars compacta (SNpc) of saline‐ or 1‐methyl‐4‐phenyl‐1,2,3,6‐tetrahydropyridine (MPTP)‐injected AQP4^+/+^ or AQP4^−/−^ mice. Scale bar = 50 μm. Mean ± SEM, n = 5. *P < 0.05, ***P < 0.001 compared with PBS‐treated AQP4^−/−^ neurons

In neurons, α‐syn is abundant.^42^ Both our qRT‐PCR (Figure 3A) and Western blotting (Figure 3B,C) results showed that even without MPP^+^ treatment, neurons from AQP4^−/−^ mice expressed much higher levels of α‐syn than did neurons from AQP4^+/+^ mice. After MPP^+^ treatment, α‐syn mRNA expression was significantly increased in AQP4^−/−^ neurons but not in AQP4^+/+^ neurons. Compared with PBS‐treated AQP4^−/−^ neurons, MPP^+^‐treated AQP4^−/−^ neurons exhibited an increasing trend in the expression of α‐syn protein.

The midbrain sections of both AQP4^+/+^ and AQP4^−/−^ mice were examined for TH and α‐syn staining after MPTP treatment. Figure 3D shows a similar level of TH staining in AQP4^+/+^ and AQP4^−/−^ mouse midbrains without MPTP treatment. After MPTP treatment, TH staining was decreased in both AQP4^+/+^ and AQP4^−/−^ mouse midbrains, and TH staining was more significantly decreased in AQP4^−/−^ mice. Although α‐syn staining was much stronger in AQP4^−/−^ mice with or without MPTP treatment, α‐syn staining was slightly increased in both AQP4^+/+^ and AQP4^−/−^ mice. In addition, partial co‐staining of TH and α‐syn was observed under both basal and MPTP‐treatment conditions.

These results suggest that α‐syn was mainly derived from neurons. However, in addition to neurons, there are many other sources of α‐syn.

### 3.4 AQP4^−/− ^mouse midbrains expressed higher levels of α‐syn with or without acute or chronic MPTP treatment

**Figure 4:**
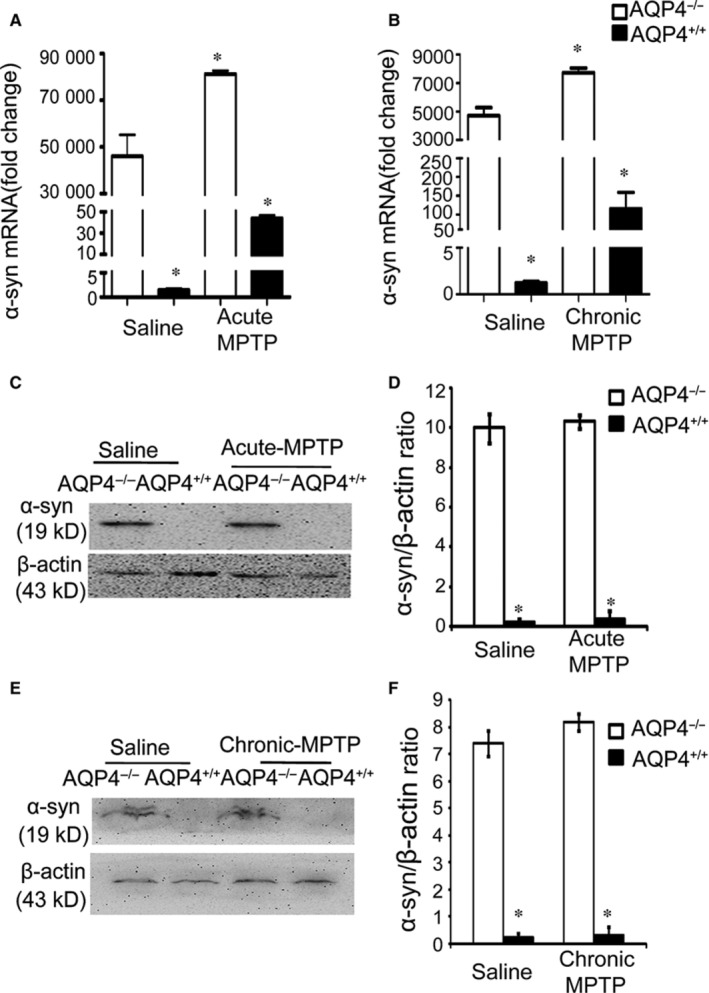
Expression of α‐synuclein (α‐syn) in the midbrains of AQP4^+/+^ and AQP4^−/−^ mice after acute or chronic 1‐methyl‐4‐phenyl‐1,2,3,6‐tetrahydropyridine (MPTP) intoxication. Quantitative real‐time PCR (Qrt‐PCR) analysis of α‐syn mRNA expression in AQP4^+/+^ and AQP4^−/−^ mice after acute (A) or chronic (B) MPTP administration. The mRNA expression was measured individually and normalised to glyceraldehyde 3‐phosphate dehydrogenase (GAPDH). Western blot analysis of α‐syn expression in both AQP4^+/+^ and AQP4^−/−^ mouse midbrains after either acute (C,D) or chronic (E,F) MPTP treatment. β‐Actin served as a loading control. The histograms represent the normalized levels of α‐syn expressed as a ratio. Data represent the mean ± SEM from five mice per group and are representative of three independent experiments. *P < 0.05 compared with saline‐treated AQP4^−/−^mice

To further investigate the possible role of α‐syn in the more severe neuronal pathology in MPTP‐treated AQP4^−/−^ PD mice, we assessed the expression levels of α‐syn mRNA and protein in the midbrains of AQP4^+/+^ and AQP4^−/−^ mice after acute or chronic MPTP intoxication. AQP4^−/−^ mice expressed very high levels of α‐syn at the mRNA (Figure 4A,B) and protein levels (Figure 4C‐F) even without MPTP treatment. After acute (Figure 4A) or chronic (Figure 4B) MPTP induction of PD, although α‐syn mRNA was further increased in AQP4^−/−^ mouse midbrains, α‐syn protein levels did not show any detectable increase in either AQP4^+/+^ or AQP4^−/−^ mouse midbrains in acute (Figure 4C,D) or chronic (Figure 4E,F) MPTP‐induced PD mouse models. These data suggested that the presence of α‐syn may be insufficient to induce and/or maintain more severe neuronal pathology in AQP4^−/−^ PD mice.

### 3.5 MPTP treatment failed to increase TGF‐β1 production in AQP4^−/− ^mice

**Figure 5:**
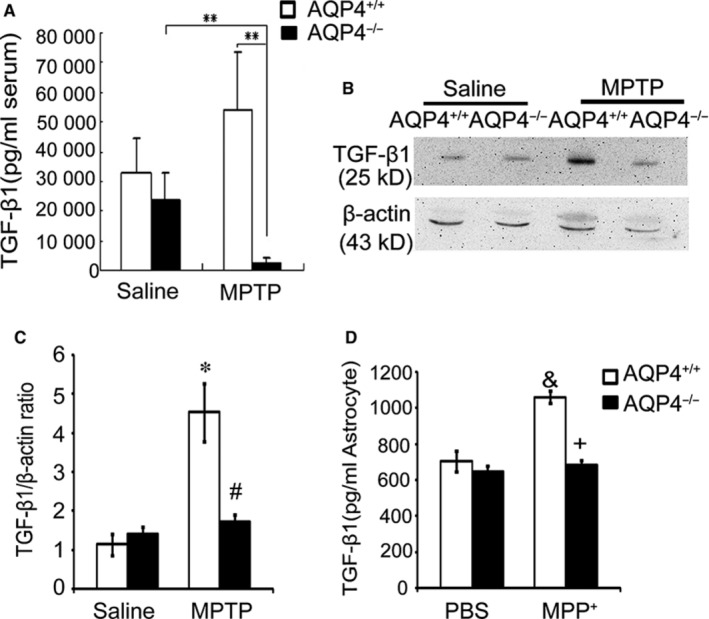
1‐methyl‐4‐phenyl‐1,2,3,6‐tetrahydropyridine (MPTP)‐treatment failed to increase transforming growth factor‐β1 (TGF‐β1) production in AQP4^−/−^ mice or in AQP4^−/−^ astrocytes. A, TGF‐β1 in the peripheral serum of AQP4^+/+^ and AQP4^−/−^ mice after acute MPTP intoxication was measured with ELISA. Data were from five mice per group and are representative of three independent experiments. B, Western blot detection of TGF‐β1 in the midbrains of AQP4^+/+^ and AQP4^−/−^ mice after acute MPTP intoxication. One representative experiment of three is shown. C, Values are presented as the mean ± SEM for five mice per group from three independent experiments and were normalized to the protein concentrations of cell extracts. D, TGF‐β1 detection by ELISA in the supernatants collected from midbrain astrocytes incubated for 48 h with 50 μmol/L of MPP^+^. **P < 0.01 compared with MPTP‐treated AQP4^−/−^ mice; *P < 0.05 compared with saline‐treated AQP4^+/+^ mice; #*P* < 0.05 compared with MPTP‐treated AQP4^+/+^ mice; &*P* < 0.01 compared with phosphate buffered saline (PBS)‐treated AQP4^+/+^ astrocytes; +*P* < 0.01 compared with MPP^+^‐treated AQP4^+/+^ astrocytes

Transforming growth factor‐β1 plays a critical role in the down‐regulation of microglial responses to minimize brain inflammation and efficiently restricts the exacerbation of brain damage in both human PD and MPTP‐induced mouse models of PD.^43^, ^44^, ^45^, ^46^, ^47^, ^48^ AQP4^+/+^ and AQP4^−/−^ mice showed similar levels of serum TGF‐β1 without MPTP treatment. 1‐methyl‐4‐phenyl‐1,2,3,6‐tetrahydropyridine treatment resulted in an increase in serum TGF‐β1 in AQP4^+/+^ mice only (Figure 5A). Consistently, after MPTP treatment, a more significant increase in the level TGF‐β1 was shown in the midbrain of AQP4^+/+^ but not AQP4^−/−^ mice (Figure 5B,C).

Midbrain TGF‐β1 is mainly expressed by microglia and astrocytes.^49^, ^50^, ^51^ Since AQP4 was expressed in astrocytes but not in microglia,^28^, ^35^, ^36^, ^37^ we further investigated the expression of TGF‐β1 in astrocytes. The results (Figure 5D) showed that compared with PBS treatment, MPP^+^ treatment resulted in increased TGF‐β1 levels in AQP4^+/+^ but not in AQP4^−/−^ astrocytes. These results indicated that AQP4 deficiency in mouse astrocytes resulted in the failure to increase TGF‐β1 production in response to MPP^+^ treatment; this issue may at least partially contribute to the more severe microgliosis and neuronal damage.

### 3.6 Injection of TGF‐β1 in the striatum significantly reduced neuronal damage and microglial activation in MPTP‐treated AQP4^−/−^ mice

**Figure 6:**
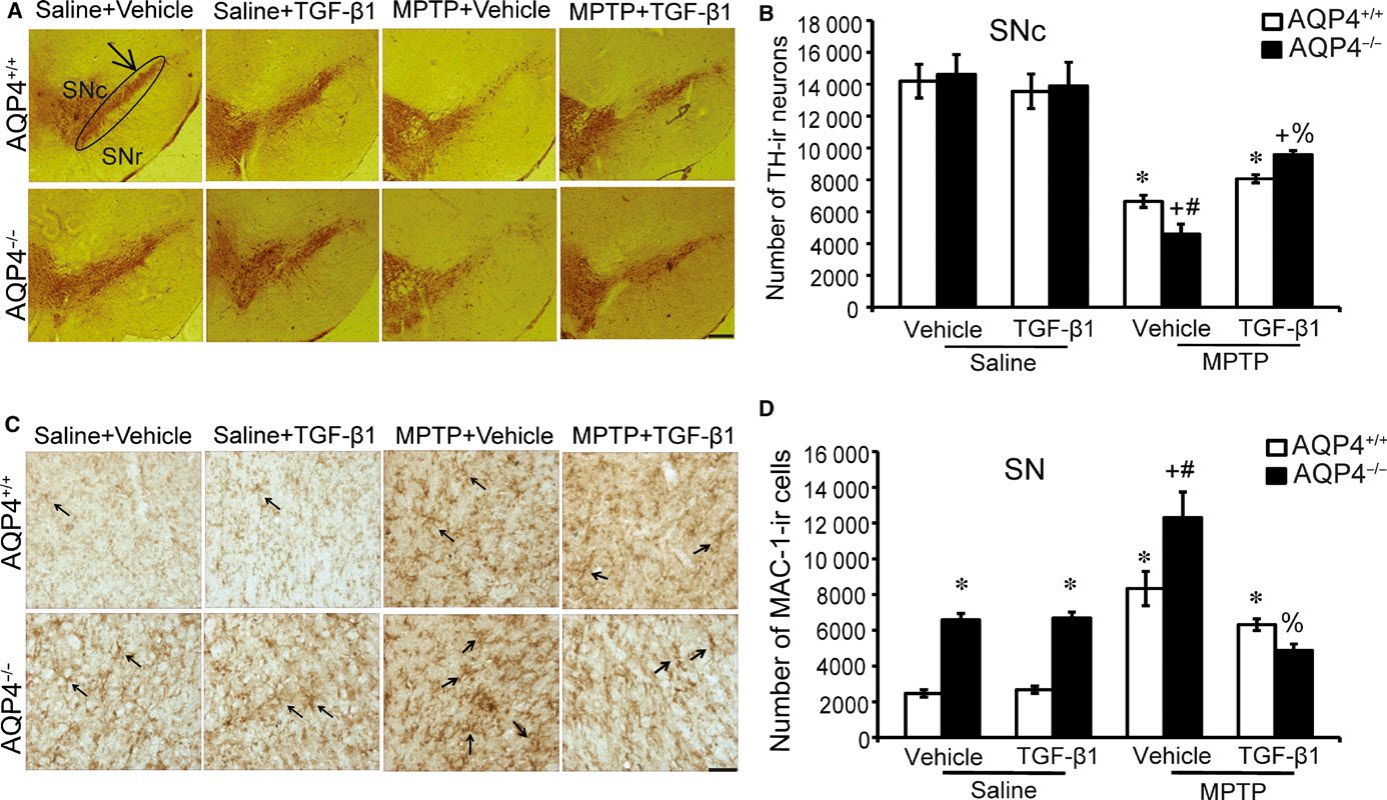
Intrastriatal injection of 50 ng of recombinant human transforming growth factor‐β1 (rhTGF‐β1) effectively attenuates 1‐methyl‐4‐phenyl‐1,2,3,6‐tetrahydropyridine (MPTP)‐induced microglia activation and dopaminergic neuronal death. In each of three independent experiments, 12 AQP4^+/+^ and AQP4^−/−^ mice per group were injected ip four times with MPTP (20 mg/kg) or equal volumes of saline as a control. After the last saline or MPTP injection, 12 mice in each group were randomly divided into two groups (six mice in each group) to receive vehicle, and the other half received a TGF‐β1 intrastriatal injection (1.5 µg/kg), respectively. A, Immunostaining for tyrosine hydroxylase‐positive cells (TH^+^) dopaminergic neurons (DNs) in the substantia nigra pars compacta (SNpc) from mice. The black circle indicates the TH^+^ (SNpc) area. B, Quantitation of TH^+^ neurons in the SNpc (black circle). C, Immunostaining for MAC‐1 microglia are labeled with black arrows (magnification: 200×). D, Quantitation of MAC‐1 microglia in the SN (black circle). Data represent the mean ± SEM for six mice per group and are representative of three independent experiments. *P < 0.01, compared with saline/vehicle‐injected AQP4^+/+^ mice and saline/TGF‐β1‐injected AQP4^+/+^ mice; +*P* < 0.01, compared with saline‐injected AQP4^−/−^ mice and saline/TGF‐β1‐injected AQP4^−/−^ mice; #*P* < 0.05, compared with MPTP/vehicle‐injected AQP4^+/+^ mice and MPTP/TGF‐β1‐injected AQP4^+/+ ^mice; %*P* < 0.05, compared with MPTP/vehicle‐injected AQP4^−/−^ mice and MPTP/TGF‐β1‐injected AQP4^−/−^ mice

To investigate whether lower levels of TGF‐β1 may be responsible for the more severe inflammation and pathology in AQP4^−/−^ mouse brains, we increased TGF‐β1 in AQP4^−/−^ mice by stereotactic injection 24 hours after the last MPTP injection. The results in Figure 6A and B show that there were no significant differences in the stereological counts of TH^+^ SNpc DNs between saline‐injected AQP4^−/−^ mice and their AQP4^+/+^ littermates without MPTP treatment. After treatment with MPTP, there were remarkably greater losses of TH^+^ neurons in the SNpc in AQP4^−/−^ mice than in AQP4^+/+^ mice. However, TGF‐β1 stereotactic injection efficiently decreased the loss of TH^+^ DNs in both AQP4^+/+^ and AQP4^−/−^ mice. More importantly, TGF‐β1 rescued many more TH^+^ DNs in AQP4^−/−^ mice.

Since MPTP induces a robust microglial response,^52^ we characterized microglial activation in mice after MPTP treatment. Mac‐1 immunostaining showed significantly fewer microglia in MPTP‐injected AQP4^+/+^ and AQP4^−/−^ mice after receiving TGF‐β1 (Figure 6C,D). In addition, TGF‐β1 attenuated more Mac‐1 immunostaining cells in AQP4^−/−^ mice.

These data suggest that TGF‐β1 more effectively attenuates MPTP‐induced microglial activation and dopaminergic neuronal death.

### 3.7 In vitro increases or decreases in TGF‐β1 significantly regulated mouse brain homogenate‐stimulated BV‐2 activation

**Figure 7:**
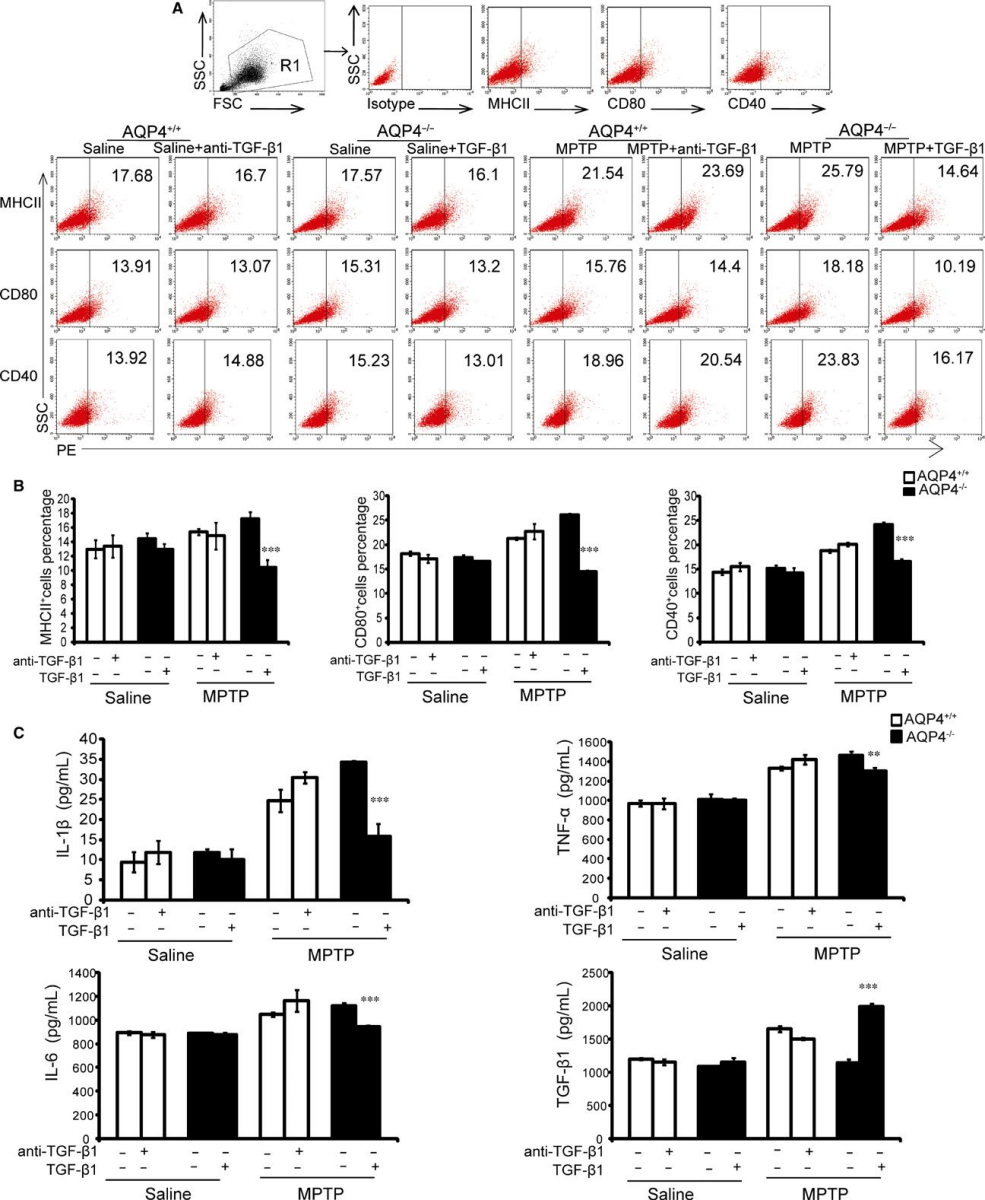
Pre‐treatment with transforming growth factor‐β1 (TGF‐β1) attenuates MPTP‐treated AQP4^−/−^ mouse brain homogenate‐induced BV‐2 activation. BV‐2 cells were pre‐treated with either TGF‐β1 or anti‐TGF‐β1 for 60 min, followed by the addition of brain homogenates from saline or 1‐methyl‐4‐phenyl‐1,2,3,6‐tetrahydropyridine (MPTP)‐injected AQP4^−/−^ or AQP4^+/+^ mice for 24 h. A, Cells were stained with anti‐MHCII‐PE, anti‐CD80‐PE or anti‐CD40‐PE. The FACScan profiles of a representative from three experiments are shown. B, Histograms are expressed as the percentage of surface marker positive cells in the total cell population. Representative histograms obtained by flow cytometry analysis. Interleukin‐1β (IL‐1β), tumour necrosis factor‐α (TNF‐α), interleukin‐6 (IL‐6), and transforming growth factor‐β1 (TGF‐β1) levels (C) were determined using ELISA. The data are expressed as the mean ± SD, n = 6. *P < 0.05, **P < 0.01, ***P < 0.001, compared with MPTP‐injected AQP4^−/−^ mice

To further confirm that the lower level of TGF‐β1 in AQP4^−/−^ mouse midbrains contributed to more severe hyperactive microglial cell responses, we added TGF‐β1 to the AQP4^−/−^ mouse midbrain homogenate or used anti‐TGF‐β1 to neutralise TGF‐β1 in the AQP4^+/+^ mouse midbrain homogenate. The addition of TGF‐β1 to the brain homogenate from MPTP‐treated AQP4^−/−^ mice resulted in a significant decrease in costimulatory molecules MHCII, CD80, and CD40 (Figure 7A,B), as well as pro‐inflammatory cytokines IL‐1β, TNF‐α and IL‐6 in BV‐2. In contrast, adding TGF‐β1 neutralizing antibodies to the brain homogenates from MPTP‐treated AQP4^+/+^ mice resulted in an increasing trend in pro‐inflammatory cytokines IL‐1β, TNF‐α, and IL‐6, but there were no significant differences (Figure 7C). These data further suggested that the lower TGF‐β1 level might be one of reasons in AQP4^−/−^ mice contributed to stronger microglial activation, which might subsequently result in more dopaminergic neuronal death and more severe PD disease after MPTP intoxication, and certain other factor(s) in brain homogenates might also be responsible for the more microgliosis and neuronal damage after MPTP treatment.

## 4 DISCUSSION

Aquaporin‐4 is a predominant water channel protein in mammalian brains that is mainly localized to the astrocyte plasma membrane.^25^ In our previous study, AQP4^−/−^ mice showed significantly stronger microglial responses and exhibited significantly more severe neuronal pathology after administration of MPTP.^28^ However, the mechanisms remain unclear. In this study, for the first time, we revealed that the significantly reduced TGF‐β1 in AQP4^−/−^ mice may lead to hyperactive microglial neuroinflammatory responses and enhance the loss of DNs by MPTP‐induced neurotoxicity.

Post‐mortem studies and animal experiments linked microglia‐mediated neuroinflammation to losses of TH^+^ neurons and the pathogenesis of PD. Overactivation of microglial cells may cause severe brain tissue damage in various neurodegenerative diseases.^53^, ^54^ However, many studies have indicated that AQP4 is not expressed on Mac‐1^+^ microglia. It might be helpful to potentially exclude the possibility of AQP4 deficiency directly leading to differential microglial responses and neuronal damage between AQP4^+/+^ and AQP4^−/−^ mice after MPTP treatment. Supportively, our study further demonstrated that certain different factor(s) in brain homogenates from AQP4^+/+^ and AQP4^−/−^ mice might be responsible for the different levels of microgliosis (differential expression of CD80 and CD40 surface molecules and pro/anti‐inflammatory cytokines) and neuronal damage after MPTP treatment. The PD protein α‐syn may play a role in microglial activation in the substantia nigra in PD and the levels of MHCII, a classical pro‐inflammatory molecule.^38^, ^55^, ^56^ However, in this study, after MPTP treatment, AQP4^+/+ ^mice showed significant neuronal pathology but only very low levels of α‐syn. In addition, the level of endogenous α‐syn was much higher in AQP4^−/− ^mice than in AQP4^+/+^ mice without MPTP treatment. Thus, these results indicate that high levels of α‐syn alone may be insufficient to induce neuronal pathology. Instead, previous studies showed that α‐syn may play an adjunctive role in PD by enhancing microglial activation‐mediated neuronal pathology, which is triggered by certain factors that are necessary for the onset of PD (eg, MPTP). Previous studies^57^ and our studies suggested the high level of α‐syn may make AQP4^−/−^ mice more susceptible to MPTP, but the mechanism still needs to be further improved. A decreased number of CD4^+^CD25^+^ regulatory T (Treg) cells promote the increased severity of PD in AQP4‐deficient mice.^28^ TGF‐β1 signalling is required for the generation of the peripheral Treg cell subset by inducing the expression of the transcription factor fork head box P3 (Foxp3).^58^ In addition, TGF‐β1 plays a critical role in the down‐regulation of microglial responses by suppressing the activation, proliferation and production of IL‐1, IL‐6, and TNF‐α, thereby minimizing brain inflammation.^43^ Moreover, TGF‐β1 elicits the neurotrophic activity of glial cell‐derived neurotrophic factor (GDNF) and contributes to the survival of midbrain DNs to protect against the toxic effects of MPP^+^.^59^ In this study, for the first time, we showed that AQP4 deficiency in mice resulted in the failure to increase TGF‐β1 production in the midbrain and peripheral blood after MPTP treatment; these changes might account for the hyperactive microglial neuroinflammatory responses and enhanced loss of DNs by MPTP‐induced neurotoxicity. Our observation appears to support the notion that reduced TGF‐β1 signalling in the striatum contributes to the loss of DNs in the substantia nigra.^48^

The primary cell types expressing TGF‐β1 mRNA in the adult brain are microglia and astrocytes.^49^, ^51^ According to previous research, astrocytes but not microglia express AQP4.^25^, ^28^, ^35^, ^36^, ^37^ In this study, we further demonstrated that MPP^+^ treatment failed to increase TGF‐β1 production in AQP4^−/−^ astrocytes. However, since TGF‐β1 can be produced by cells other than astrocytes in the brain, further studies are needed to explore how AQP4 controls astrocytes and/or other cells to generate TGF‐β1.

## 5 CONCLUSIONS

Our study illustrated that the TGF‐β1 production in astrocytes was impaired in AQP4^−/−^ mice; this alteration may contribute to the hyperactive microglial neuroinflammatory responses and subsequent enhancement of the loss of DNs by MPTP‐induced neurotoxicity.

## CONFLICT OF INTEREST

The authors declare no competing interests.

## AUTHORS’ CONTRIBUTIONS

C.S. and G.H. designed the project and provided funds to develop it. Experiments were mostly performed by X.X., W.W.Z. and J.F.Z. under the direct supervision of C.S. and G.H. X.J.C., Z.P.X. and S.Z. contributed to the critical interpretation of the data. X.X. contributed to the first draft of the manuscript, which was finally completed by C.S. and revised by all the authors of the manuscript. X.X. and X.J.C. edited the final text according to the journal's style. Specific contributions to figures are as follows: (a) In Figures 1, 2, 3, 4, X.X. and W.W.Z. generated the data; X.X. assembled the figure and edited the graphics. (b) In Figures 5, 6, 7, X.X. and J.F.Z. generated the data under the supervision and advice of X.J.C. and S.Z. X.X. assembled the figure and Z.P.X. edited the graphics.
